# Size selection of intrahepatic lesions for cryoablation contributes to abscopal effect and long-term survival in patients with liver metastatic melanoma receiving PD-1 blockade therapy

**DOI:** 10.1007/s00262-024-03637-1

**Published:** 2024-03-02

**Authors:** Lujun Shen, Hongtong Tan, Juan Nie, Yiquan Jiang, Gulijiayina Nuerhashi, Han Qi, Fei Cao, Chunyong Wen, Shuanggang Chen, Tianqi Zhang, Wei Zheng, Peng Liu, Ying Liu, Tao Huang, Dandan Li, Xiaoshi Zhang, Weijun Fan

**Affiliations:** 1 Department of Minimally Invasive Interventional Therapy, Sun Yat-Sen University Cancer Center, Guangzhou, 510060 People’s Republic of China; 2 State Key Laboratory of Oncology in South China, Guangdong Provincial Clinical Research Center for Cancer, Sun Yat-Sen University Cancer Center, Guangzhou, 510060 People’s Republic of China; 3 Department of Research & Education, Guangzhou Concord Cancer Center, Guangzhou, 510054 People’s Republic of China; 4 Department of Oncology, Yuebei People’s Hospital, Shaoguan, 511100 People’s Republic of China; 5 Chongqing Emergency Medical Center, Chongqing University Central Hospital, Chongqing, 400000 People’s Republic of China; 6 Department of Oncology, General Hospital of Southern Theater Command, Guangzhou, 510060 People’s Republic of China; 7 Department of Biological Therapy Center, Sun Yat-Sen University Cancer Center, Guangzhou, 510060 People’s Republic of China

**Keywords:** Melanoma, Liver metastasis, Cryoablation, PD-1 blockade therapy, Abscopal effect

## Abstract

**Objectives:**

In this study, we aimed to examine parameters of cryoablation, tumor characteristics, and their correlations with distant tumor response and survival of liver metastatic melanoma patients receiving cryoablation and PD-1 blockade (cryo-PD-1) combination treatment.

**Materials and methods:**

A retrospective study was conducted among 45 melanoma patients who received combined PD-1 blockade therapy and cryoablation for liver metastasis from 2018 to 2022. Cox regression was utilized to determine the associations between factors and overall survival (OS). Changes in cytokines and immune cell compositions in peripheral blood samples following the combined treatment were investigated, along with their correlations with treatment response.

**Results:**

The mean cycle of cryo-PD-1 combination treatment was 2.2 (range, 1–6), and the 3-month overall response rate (RECIST 1.1 criteria) was 26.7%. Of the 21 patients who failed previous PD-1 blockade therapy after diagnosis of liver metastasis, 4 (19.0%) achieved response within 3 months since combination treatment. The diameter of ablated lesion  ≤ 30 mm, metastatic organs  ≤ 2, and pre-treatment LDH level  ≤ 300 U/L were independent prognostic factors for favorable OS. Further analysis showed patients with intrahepatic tumor size of 15–45 mm, and ablated lesion size of  ≤ 30 mm had significantly higher 3-month response rate (42.9% vs 12.5%; *P* = 0.022) and survival time (30.5 vs 14.2 months; *P* = 0.045) than their counterparts. The average increase in NLR among patients with ablated tumor size of  ≤ 3 cm and  > 3 cm were 3.59 ± 5.01 and 7.21 ± 12.57, respectively. The average increase in serum IL-6 levels among patients with ablated tumor size of  ≤ 3 cm and  > 3 cm were 8.62 ± 7.95 pg/ml and 15.40 ± 11.43 pg/ml, respectively.

**Conclusion:**

Size selection of intrahepatic lesions for cryoablation is important in order to achieve abscopal effect and long-term survival among patients with liver metastatic melanoma receiving PD-1 blockade therapy.

**Supplementary Information:**

The online version contains supplementary material available at 10.1007/s00262-024-03637-1.

## Introduction

Liver metastasis remains a clinical challenge for immune checkpoint inhibitor therapy in melanoma, with an estimated response rate ranging from 3 to 10% [1]. Recent translational studies have dissected the mechanism of an immune suppression effect of liver metastasis [2]. Consequently, there is urgent demand for effective interventions to overcome the immune suppressive effect of liver metastasis [3].

Cryoablation is recognized for its potential to trigger strong anti-tumor immune responses [4]. The low temperature by cryoablation can result in direct cell death, leading to tumor antigen release and tilting the immune balance toward Th1 immune response [5]. A recent ambispective cohort study showed that combined PD-1 blockade therapy and cryoablation of liver metastasis in patients with metastatic melanoma could achieve an overall response rate (ORR) of 26.7% of unablated lesions, with a significant increase in NK cells and a marginal decrease in regulatory T cells in the peripheral blood [6]. Although cryoablation has been demonstrated to have the potential to elicit a strong abscopal effect, only a small proportion of patients can benefit from this combination treatment [7, 8]. Identifying patients who can benefit from combination therapy is essential in maximizing its treatment effect.

Concurrently, recent studies have reported an increasing concern regarding the potential inflammatory response induced by ablation, which may, in turn, compromise the anti-tumor immune response [9, 10]. This indicates that ablating multiple lesions in one session or treating excessively large tumors during cryoablation could lead to immune suppression. Given the controversy regarding whether the cryoablation could induce or impair anti-tumor immune response, it is imperative to refine the procedural criteria for ablation in combined treatment. This refinement may yield valuable insights for understanding the nuanced factors influencing the immune response and maximizing treatment outcomes. Therefore, we conducted the Cryo-Fire-001 study, aiming to examine parameters of cryoablation, tumor characteristics, and their correlations with distant tumor response and survival of liver metastatic melanoma patients receiving cryoablation and PD-1 blockade combination treatment.

## Methods

### Patients

This study retrospectively reviewed the medical records of a consecutive series of 120 melanoma patients with multiple hepatic metastases in Sun Yat-sen University Cancer Center (SYSUCC) from September 1, 2018, to December 30, 2021. The inclusion criteria for this study were: (a) histological diagnosis of melanoma; (b) with multiple liver metastases; (c) received combined cryoablation of liver metastases and PD-1 blockade therapy; (d) had measurable non-ablated lesion for response assessment during the combination treatment; (e) performance score of 0 or 1 based on the Eastern Cooperative Oncology Group (ECOG) Performance Scale; and (f) no active or a documented history of autoimmune disease. The exclusion criteria were: (a) received PD-1 blockade therapy before identification of liver metastases and (b) with other malignancies. The Hospital Ethics Committee of SYSUCC approved this study (B2022-693-01), which waived the need for written informed consent based on the retrospective nature of the study. The Chinese Trial Register (ChiCTR) identifier was ChiCTR2300069319. A total of 45 patients were enrolled. Our previous ambispective cohort study evaluating the efficacy of cryoablation combined with PD-1 blockade therapy included 15 liver metastatic melanoma patients [6]; these patients were also included in the analysis with an aim of identifying the factors in cryoablation that determines the success of this combination therapy.

### Cryoablation and PD-1 blockade therapy

The combined cryoablation and PD-1 blockade therapy included two stages: the initial combined stage and the subsequent PD-1 blockade maintenance stage. During the combined stage, patients were recommended to receive four cycles of combination treatment. The cryoablation of liver metastases had to be performed within a week of PD-1 blockade therapy. The combined treatment was repeated at an interval of 3 weeks and could be terminated due to the absence of intrahepatic viable lesions, intolerable toxicity, confirmed disease progression, or patients’ requests. If intrahepatic tumors did not progressed, cryoablation would be recommended to continue until all lesions with diameter larger than 5 mm and in safe location had been ablated. In the PD-1 blockade maintenance stage, the anti-PD-1 antibody was administered every 3 weeks until disease progression, presence of unacceptable toxicity, or loss to follow-up.

Cryoablation was performed using Visual-ICE™ System (Galil Medical, Israel). For each procedure, 1–4 intrahepatic lesions with the longest diameter of  < 7 cm were ablated; for multiple target tumors larger than 3 cm, no more than two lesions were ablated in a single procedure. Targeted lesions were chosen and treated with full ablative intent at the direction of interventional radiologists (W.F., W.L., and L.S. with 18, 16, and 10 years of experience in percutaneous cryoablation, respectively) based on critical technical factors, such as away from large intrahepatic vessels and ease of access. If intrahepatic tumors did not progress, cryoablation was recommended to continue until all intrahepatic lesions with a diameter larger than 5 mm and in a safe location ablated. Under normal circumstances, 2–3 cryoprobes are used for lesions with a diameter of  < 3 cm, 4–6 cryoprobes are used for lesions with a diameter of 3–5 cm, and 7–8 cryoprobes are used for lesions with a diameter of  > 5 cm. The number of cryoprobes used may be reduced when special situations (e.g., abdominal hemorrhage and pneumothorax) happened, which were judged by the interventional radiologist.

During the procedure, local anesthesia with 2% lidocaine was implemented. The cryoprobes were planned and inserted into the target tumor under CT guidance (Brilliance CT Big Bore; PHILIPS); two 10-min freezing cycles, separated by a passive 10-min and an active 2-min thawing session, were performed. Overlapping ablations with the same ablation parameters were conducted when needed. The technical success of cryoablation was defined as the ice ball extending at least 5 mm beyond the tumor’s boundaries. The vital signs of each patient were monitored throughout the procedure. A non-contrast CT scan was performed to identify any early complications after the removal of cryoprobes. Routine blood tests and biochemical tests were obtained within 24 h after the treatment.

For the administration of anti-PD-1 antibody (pembrolizumab/toripalimab), a homogeneously mixed solution of anti-PD-1 antibody and 100-ml normal saline was injected slowly, which took 30–60 min.

### Other treatments

Concurrent additional treatments including anti-angiogenic therapy (lenvatinib/apatinib/axitinib) and chemotherapy (paclitaxel–albumin/temozolomide) were adopted based on the shared decision made together by patients and a multidisciplinary team consisting of oncologists, interventional radiologists, radiologists, and surgeons.

### Ablation parameters and variables

The parameters of the first cryoablation included the largest diameter of intrahepatic lesions ablated, number of needles, number of lesions ablated, and time from identification of liver metastasis to first cryoablation. Previous PD-1 blockade therapy (absent/present) was defined as whether or not to use PD-1 blockade therapy before combined treatment after identification of liver metastasis. The timing of the combined anti-PD-1 antibody was dichotomized into before cryoablation and after cryoablation based on the time sequence between the first cryoablation and its nearest administration of anti-PD-1 antibody.

### Immune correlative studies

A proportion of the patients agreed to conduct blood sampling to monitor the changes in serum immune markers. The percentage of lymphocytes subsets, including T cells (CD3+), B cells (CD19+), NK cells (CD3-CD16+CD56+), cytotoxic T-cell (CD3+CD8+), and regulatory T cells (CD4+CD25+), were tested by multicolor flow cytometry. Levels of Th1 and Th2 cytokines, including IL-2, IL-4, IFN-γ, IL-6, and IL-10, were detected using immunofluorescence analysis, with a detection limit from 2.5 pg/ml to 5000 pg/ml.

### Follow-up and endpoints

Patients were followed up every 6–12 weeks through contrast-enhanced MRI/CT scans during the combination treatment. The primary endpoint was overall survival (OS), which was defined as the time from the start of combination treatment to death by any causes. The secondary endpoint was objective response at 3 months. Exploratory endpoints were changes in cytokines and immune cell compositions in peripheral blood samples. Objective response for distant tumors was evaluated in lesions not subject to ablation, and the response assessments were completed every 8–12 weeks by two experienced radiologists (W.Z. and L.S., with 10 and 8 years experience in medical imaging, respectively) using Response Evaluation Criteria in Solid Tumors, version 1.1 (RECIST v1.1) [11].

### Statistical analysis

Efficacy by response rate was reported as percentages. Pearson Chi-square test was used to compare categorical variables by groups, and Fisher’s exact test was utilized when the expected count of any cell in the contingency table was less than 5. X-tile software, which can optimize the significance of the split between Kaplan–Meier survival curves, was used to determine the optimal cutoffs of immune correlative studies in predicting OS; the derived cutoffs were validated by log-rank tests to ensure its significance. The Cox regression model was utilized to evaluate the prognostic factors of OS. Paired t-test was used to compare the differences in serum immune indicators before and after the cryoablation, and independent samples t-test was used to analyze the differences in serum immune indicators between responders and non-responders. Chi-square test and survival analyses were done using SPSS 21.0. The sklearn module was utilized to build a decision tree model in the Python 3.6 environment, with random_state of 25, criterion of “entropy,” splitter of “random,” and max_depth equaled to 2. Based on the observation of the scatter plot describing the correlation between diameter of intrahepatic lesion, diameter of the ablated lesion, and treatment response, the covariates included were diameter of ablated lesion, diameter of intrahepatic lesion, size of intrahepatic tumor within 15–45 mm (present/absent), and size of ablated lesion  ≤ 30 mm (present/absent). A *P* value < 0.05 was considered significant.

## Results

### Patient characteristics

The baseline characteristics of the enrolled patients are listed in Table 1. The median age of the patients was 53 (range, 35–79). Fifteen patients (33.3%) had cutaneous melanoma, 20 patients (44.4%) had mucosal melanoma, 8 patients (17.8%) had uveal melanoma, and 2 (4.4%) had melanoma with unknown origin. Most patients (77.8%) had disseminated liver metastasis (> 3), and thirteen patients (28.9%) had more than two metastatic organs. Twenty-one (46.7%) patients failed initial PD-1 blockade therapy before combination therapy after the identification of liver metastasis. In terms of anti-PD-1 antibody, most patients (60.0%) used pembrolizumab, and the rest (40.0%) used toripalimab during combined treatment. Six patients (13.3%) added systemic chemotherapy (albumin–paclitaxel/dacarbazin), and eight patients (17.8%) added multikinase inhibitor (lenvatinib) during the combination treatment.

**Table 1:** Baseline characteristics of patients enrolled (*n* = 45)

Category	Cohort *N*, (%)
Age (median, range)	53 (35–79)
Sex	
Male	19 (42.2)
Female	26 (57.8)
Tumor origin	
Cutaneous	15 (33.3)
Other	30 (66.7)
Number of metastatic organs	
≤ 2	32 (71.1)
> 2	13 (28.9)
Number of intrahepatic metastases	
≤ 3	10 (22.2)
> 3	35 (77.8)
Diameter of the intrahepatic lesion (mm)	
≤ 30	26 (57.8)
> 30	19 (42.2)
LDH level	
≤ 300 U/L	30 (66.7)
> 300 U/L	15 (33.3)
Combined anti-PD-1 antibody	
Toripalimab	18 (40.0)
Pembrolizumab	27 (60.0)
Previous PD-1 blockade therapy	
Absent	24 (53.3)
Present	21 (46.7)
Chemotherapy	
Absent	39 (86.7)
Present	6 (13.3)
Anti-angiogenic agents	
Absent	37 (82.2)
Present	8 (17.8)
BRAF V600E/K mutation	
Absent or unknown	42 (93.3)
Present	3 (6.7)

The mean cycle of cryo-PD-1 combination treatment was 2.2 (range, 1–6). During the first combination treatment, 28 patients (62.2%) received PD-1 blockade therapy after cryoablation, and 17 patients (37.8%) received PD-1 blockade therapy before cryoablation. Technical success of cryoablation was achieved for the most (168/173; 97.1%) targeted intrahepatic lesions during one session, while abdominal bleeding (5/173, 2.9%) was the cause of termination of further overlapping ablation. All lesions failed to achieve complete ablation in one cryoablation session and underwent additional cryoablation sessions in the next cycle of combination treatment. A total of 5 (11.1%) patients had all intrahepatic lesions ablated during the combination treatment, and lesions outside liver served as target lesions for response assessment. The median follow-up time of the cohort was 22.5 (11–46) months, and the median OS time was 17.8 months.

### Cryoablation and distant tumor response

**Fig. 1:**
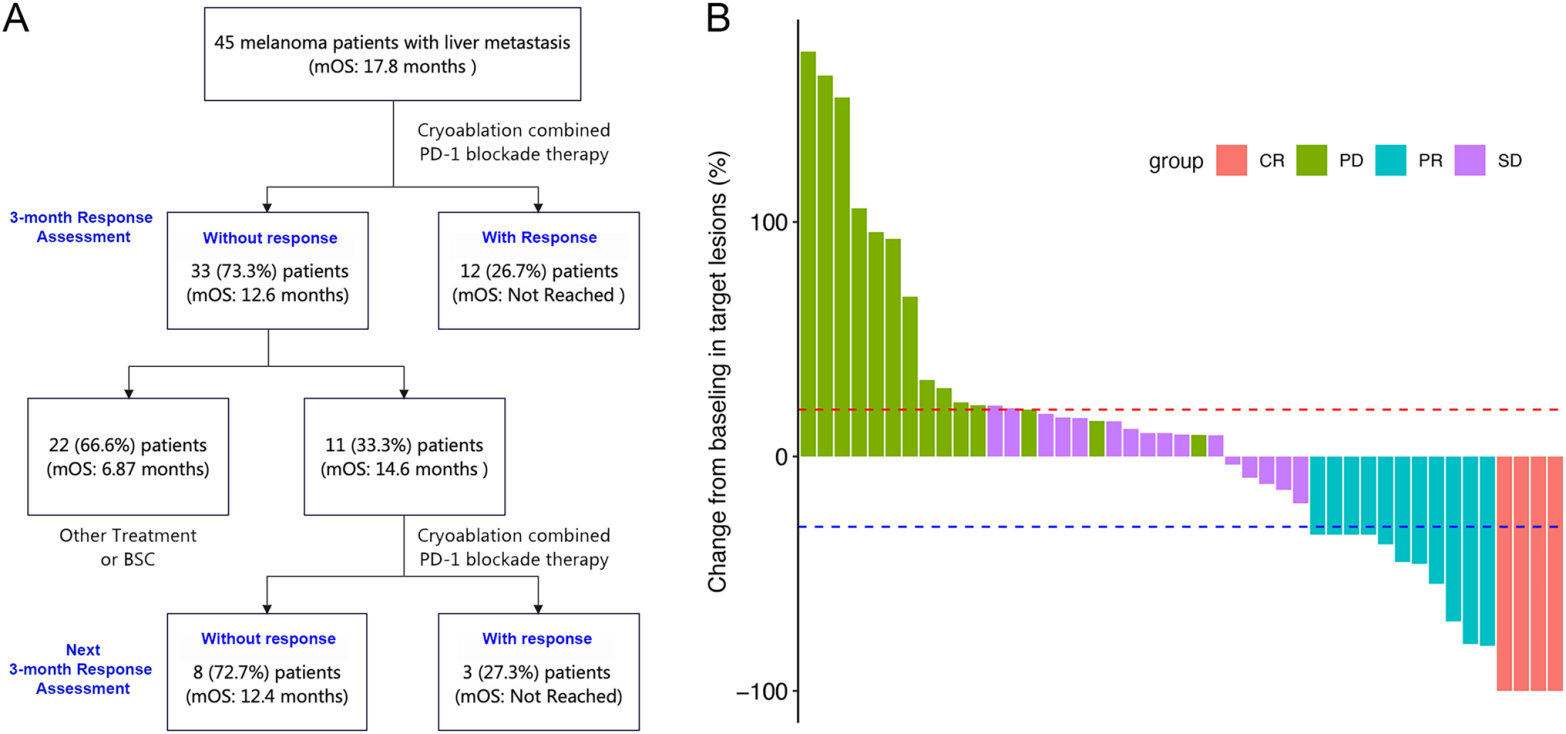
Treatment response in patients receiving cryoablation–PD-1 blockade combination therapy. The best treatment response during treatment was displayed, and 11 patients continued combination treatment without response within the first 3 months (**A**). A waterfall plot depicting maximum response of the 45 patients (**B**)

The 3-month and 6-month best overall response rates of the cohort were 26.7% (12/45) and 33.3% (15/45), respectively (Fig. 1). Of the 12 patients who achieved response within 3 months, 4 (33.3%) failed initial PD-1 blockade therapy before combination treatment. Eleven of the patients without response within the first 3 months continued the combination treatment, and 3 (27.3%) achieved PR by the end of the 6th month. Of the 21 patients who failed previous PD-1 blockade therapy after diagnosis of liver metastasis, 4 (19.0%) achieved response within 3 months since combination treatment.

The correlation between characteristics of the first cryoablation and 3-month ORR is displayed in Table 2. A marginal significant correlation was found between baseline low level of CD4 + CD25 + cells and distant tumor response (*P* = 0.078).

**Table 2:** Characteristics of first cryoablation, baseline immune markers of peripheral blood, and 3-month ORR (*n* = 45)

Category	Non-response	Response	*P* value
Diameter of lesions ablated			0.283*
≤ 30	20 (60.6)	10 (83.3)	
> 30	13 (39.4)	2 (16.7)	
Number of lesions ablated			0.524
1	20 (60.6)	6 (50.0)	
2–4	13 (39.4)	6 (50.0)	
Number of cryoablation needles			0.699*
≤ 2	24 (72.7)	10 (83.3)	
> 2	9 (27.3)	2 (16.7)	
Time from liver metastasis to first cryoablation (months)			0.325*
≤ 0.5	11 (33.3)	6 (50.0)	
> 0.5	22 (66.7)	6 (50.0)	
Timing of combined anti-PD-1 antibody			0.096*
Before cryoablation	15 (45.5)	2 (16.7)	
After cryoablation	18 (54.5)	10 (83.3)	
NLR			0.096*
≤ 2.80	18 (54.5)	10 (83.3)	
> 2.80	15 (45.5)	2 (16.7)	
CD8 + T/CD4 + CD25 + cell ratio^Ѱ^			0.458
≤ 1.60	15 (62.5)	3 (30.0)	
> 1.60	9 (37.5)	7 (70.0)	
CD3-CD16 + CD56 + cells (%)^Ѱ^			0.132
≤ 20	14 (58.3)	3 (30.0)	
> 20	10 (41.7)	7 (70.0)	
CD4 + CD25 + cells (%)^Ѱ^			0.078*
≤ 23	17 (70.8)	10 (100.0)	
> 23	7 (29.2)	0 (0.0)	
IL-6 level (pg/ml)^Ѱ^			0.134*
≤ 4.63	9 (45.0)	7 (63.6)	
> 4.63	11 (55.0)	4 (36.4)	

NLR, Neutrophil-to-lymphocyte ratio

*Fisher’s exact test

^Ѱ^A total of 34 patients have data on the level of lymphocyte subsets, and 31 patients have data on the level of Th1 and Th2 cytokines in peripheral blood before the first cryoablation

### Univariate and multivariate survival analysis

**Fig. 2:**
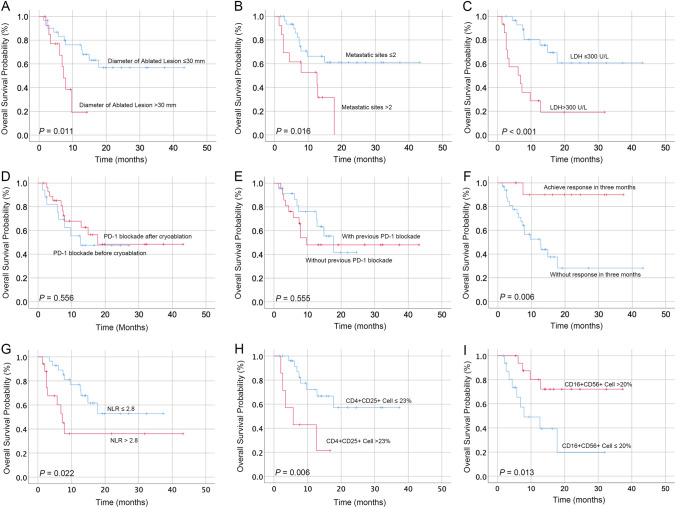
The survival curves for patients receiving cryoablation–PD-1 blockade combination therapy grouped by factors of interest. The factors included the diameter of the ablated lesion (**A**), metastatic sites (**B**), pre-treatment LDH (**C**), sequence of combined treatment (**D**), history administration of PD-1 antibody (**E**), 3-month treatment response (**F**), pre-treatment NLR (**G**), pre-treatment CD4 + CD25 + cells (**H**), and pre-treatment CD16 + Cd56 + cells (**I**). All Kaplan–Meier curves were unadjusted for clinical factors

The 1-year and 3-year OS rates of the cohort were 62.5% and 45.8%, respectively. Patients with achieved response within the first 3 months had longer OS time compared to those without response (Fig. 2). Univariate analysis showed that the number of metastatic sites (> 2/ ≤ 2), the diameter of intrahepatic tumor (> 30/ ≤ 30 mm), LDH level (> 300 U/L/ ≤ 300 U/L), pre-treatment neutrophil-to-lymphocyte ratio (NLR > 2.8/ ≤ 2.8), CD4 + CD25 + cell level (> 23%/ ≤ 23%), CD16 + CD56 + cell level (> 20%/ ≤ 20%), and diameter of the ablated lesion (> 30/ ≤ 30 mm) were significantly correlated with the OS of patients (Fig. 2; Table 3). As more than 20% of patients lacked data on lymphocyte subsets, a total of five variables, including the number of metastatic sites, the diameter of intrahepatic tumor, LDH level, pre-treatment NLR, and diameter of ablated lesion, were included in multivariate analysis. Multivariate analysis showed that metastatic organs  > 2 (HR: 3.54, 95%CI 1.32–9.49), pre-treatment LDH level  > 300 (HR:4.59, 95%CI 1.78–11.85), and diameter of ablated lesion  > 30 mm (HR: 3.27, 95%CI 1.17–9.15) were independent unfavorable prognostic factors for OS (Table 3).

**Table 3:** Univariate and multivariate analysis for melanoma patients with liver metastasis receiving cryoablation and PD-1 blockade combination treatment (*n* = 45)

Category	*n*	Univariate analysis		Multivariate analysis	
		HR (95% CI)	*P* value	HR (95% CI)	*P* value
Age (> 53/ ≤ 53)	20/25	0.85 (0.34–2.13)	0.725	–	–
Sex (female/male)	26/19	0.96 (0.38–2.40)	0.926	–	–
Tumor origin (other/cutaneous)	30/15	1.47 (0.53–4.09)	0.463	–	–
Number of metastatic sites (> 2/ ≤ 2)	13/32	2.93 (1.17–7.32)	0.022	3.54 (1.32–9.49)	0.012
Diameter of intrahepatic tumor (> 30/ ≤ 30)	20/25	2.60 (1.03–6.59)	0.044	–	–
Diameter of intrahepatic tumor (> 50/ ≤ 50)	7/38	1.15 (0.33–4.06)	0.826	–	–
Number of intrahepatic metastases (> 3/ ≤ 3)	35/10	1.66 (0.48–5.74)	0.423	–	–
LDH level (> 300 U/L/ ≤ 300 U/L)	15/30	4.87 (1.94–12.21)	0.001	4.59 (1.78–11.85)	0.002
Diameter of ablated lesion (> 30/ ≤ 30 mm)	15/30	3.40 (1.26–9.18)	0.016	3.27 (1.17–9.15)	0.024
Time from liver metastasis to cryoablation (> 0.5/ ≤ 0.5 months)	28/17	2.87 (0.95–8.69)	0.062	–	–
Anti-angiogenic agents (present/absent)	8/37	0.92 (0.21–4.01)	0.921	–	–
Chemotherapy (present/absent)	6/39	1.10 (0.32–3.80)	0.875	–	–
NLR (> 2.8/ ≤ 2.8)	28/17	2.78 (1.11–6.94)	0.029	–	–
CD16 + CD56 + cells (> 20%/ ≤ 20%)^Ѱ^	17/17	0.25 (0.08–0.81)	0.021	/	/
CD4 + CD25 + cells (> 23%/ ≤ 23%)^Ѱ^	7/27	4.47 (1.40–14.28)	0.011	/	/

^Ѱ^The pre-treatment percentage of CD3-CD16 + CD56 + cells and CD4 + CD25 + cells among immune cells were not included in the multivariate analysis due to the missing values of more than 20% among all the included patients

### Tumor diameter, ablation, and distant tumor response

Patients with larger intrahepatic tumors ablated had larger tumor sizes (*P* < 0.001) and required more cryoablation needles (*P* = 0.026) for ablation (Table S1). On the other hand, both the diameter of the intrahepatic tumor and the diameter of the ablated lesion correlated with the OS in the univariate analysis. It is, therefore, necessary to co-analyzed these two factors in order to identify the patient subgroup that benefits from the combination treatment.

**Fig. 3:**
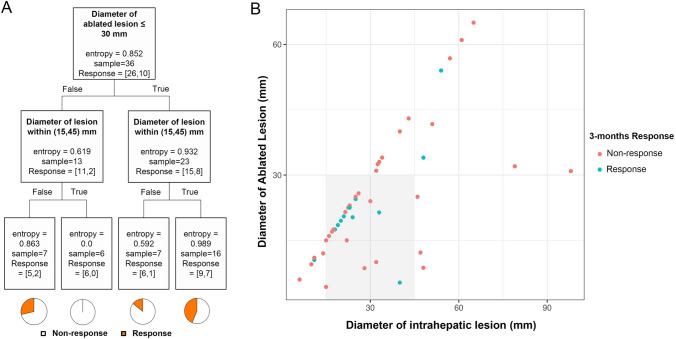
Co-analysis of the diameter of the intrahepatic lesion and the diameter of the ablated lesion to identify the patients who may benefit from the combined cryoablation and PD-1 blockade therapy. A decision tree model was built based on the training set, which reached accuracy scores of 0.722 in the training set and 0.778 in the testing set (**A**). Scatter plot showing the correlation between the largest diameter of intrahepatic lesion and diameter of the ablated lesion (**B**). Patients who achieved a 3-month response were marked blue

**Fig. 4:**
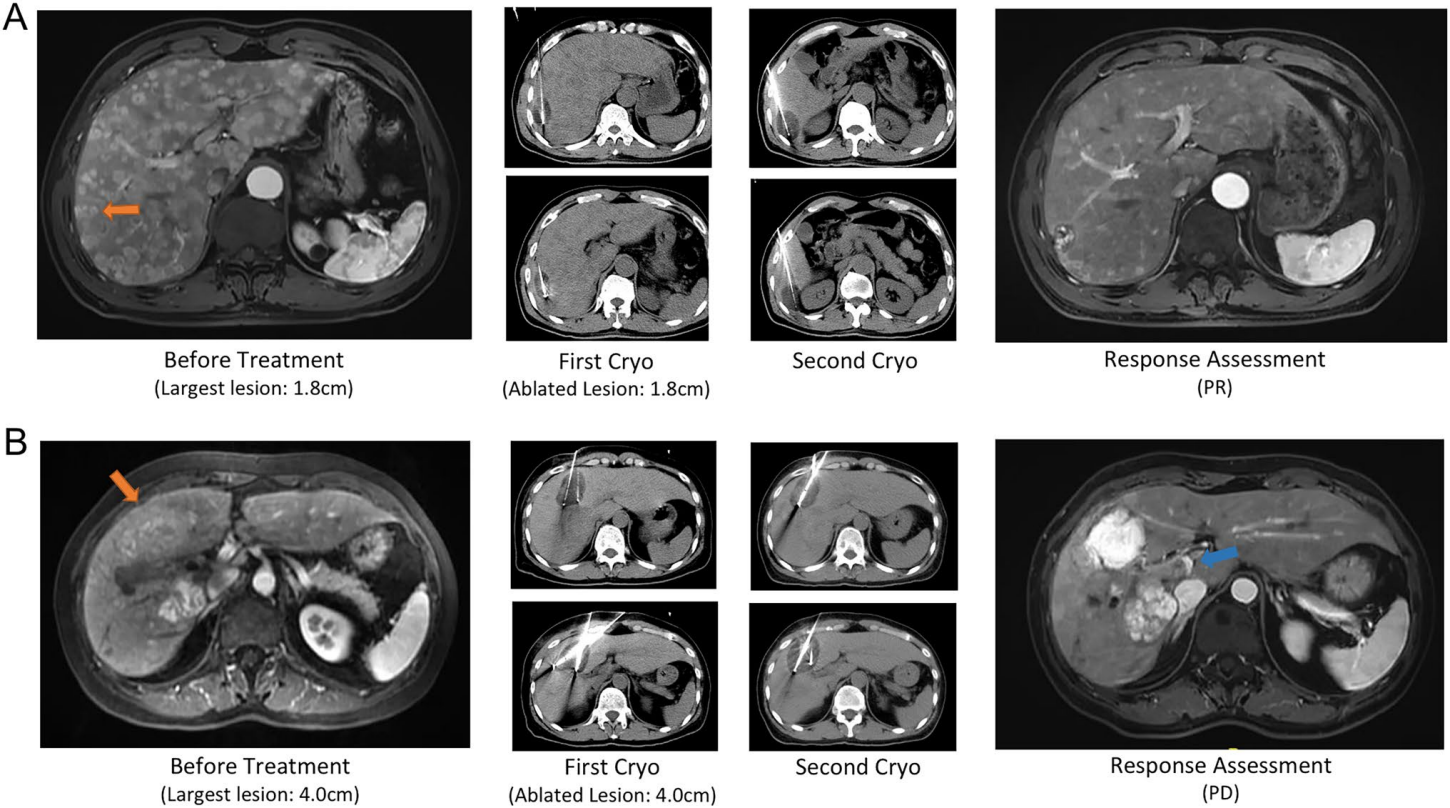
Typical cases of melanoma patients with different sizes of intrahepatic tumors receiving combined cryoablation and PD-1 blockade therapy. **A** An adult patient with cutaneous melanoma had progression in liver metastasis after two cycles of pembrolizumab therapy. Contrast-enhanced T1-weighted imaging (T1WI) showed that a major PR was achieved after two cycles of combination treatment; CR was achieved after two more cycles of pembrolizumab infusion (arrow, target lesion for the 1st cryoablation). **B** An adult patient was diagnosed of multiple liver and bone metastases after resection of uveal melanoma. She received two cycles of combination treatment while contrast-enhanced T1WI showed unablated intrahepatic tumors and protal vein tumor thrombus (PVTT) progressed continuously (orange arrow, target lesion for cryoablation and blue arrow, PVTT)

The dataset was then split into a training set and a testing set, with a test size of 20%. A decision tree model (Fig. 3A) was built in predicting 3-month treatment response (Fig. 3B). Two dichotomous variables were selected into the final model and achieved accuracy scores of 0.722 in the trainning set and 0.778 in the testing set. Patients within the square region of intrahepatic tumor size of 15–45 mm and ablated lesion size of ≤ 30 mm had significantly higher 3-month response rate (42.9% vs 12.5%; *P* = 0.022) and OS time (30.5 vs 14.2 months; *P* = 0.045) than their counterparts (Figs. 3B and 4).

### Immune changes after cryoablation and response

**Fig. 5:**
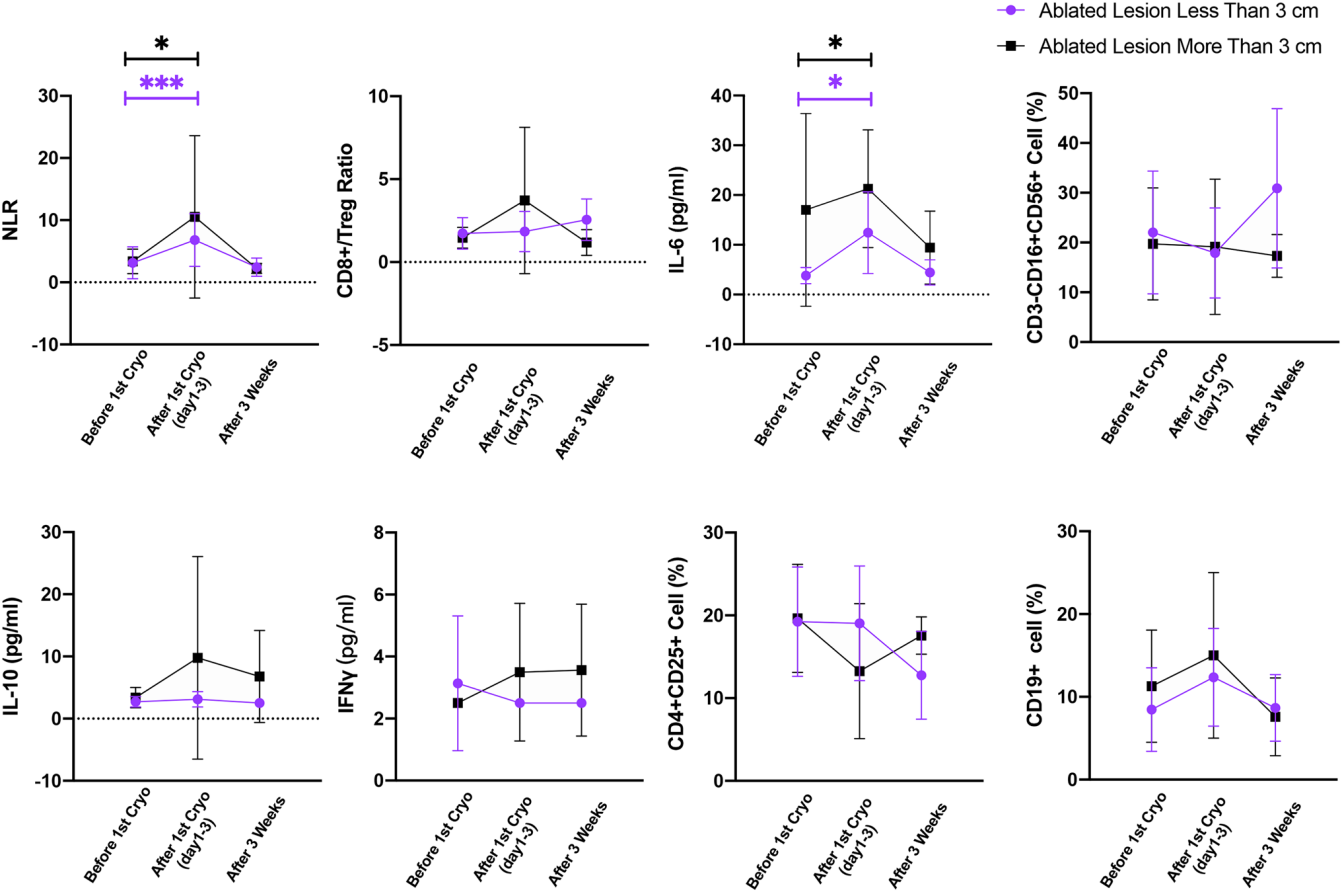
Size of ablated tumor and changes in immune correlative studies during and after the first cryoablation

A total of 34 (75.6%) patients had data on lymphocyte subsets, and 31 (68.9%) had data on cytokines since the first cryoablation. During combination treatment, cryoablation of intrahepatic lesions could lead to a transient increase in NLR and IL-6 levels in peripheral blood, and the changes could not be detected 3 weeks later (Fig. 5). The average increase in NLR among patients with ablated tumor size of ≤ 3 cm and  > 3 cm were 3.59 ± 5.01 and 7.21 ± 12.57, respectively, yet the difference did not reach statistical significance. The average increase in serum IL-6 levels among patients with ablated tumor size of  ≤ 3 cm and  > 3 cm were 8.62 ± 7.95 pg/ml and 15.40 ± 11.43 pg/ml, respectively, yet the difference did not reach significance. No significant changes in IL-10, IFN-γ, CD4 + CD25 + cells, and CD3-CD16 + CD56 + cells after the first cryoablation were found.

The changes in lymphocyte subsets, IL-6, IL-10, and IFN-γ during the first four cycles of cryo-PD-1 combination treatment were also plotted (Figure S1). The responders had a significantly lower level of CD19 + cells in the peripheral blood after the first cryoablation compared with non-responders (*P* = 0.03). Although there was a trend that responders had increased CD8/Treg ratio, CD3-CD16 + CD56 + cells, and decreased CD4 + CD25 cells after the second cryoablation, the differences did not reach statistical significance.

## Discussion

The previous studies showed that the prognosis of liver metastatic melanoma patients receiving PD-1 monotherapy was poor, with a median OS of 6–12 months since the identification of liver metastasis [12, 13]. Based on our previous study (6), this study further confirmed the efficacy of cryoablation combined with PD-1 blockade therapy in the treatment of liver metastasis of melanoma, with a median OS of 17.8 months. In investigating the prognostic factors of the combined therapy, we found that more metastatic sites, higher pre-treatment LDH levels, and larger size of ablated lesion were associated with poor overall survival after the cryoablation–PD-1 combination treatment.

Our study demonstrated a 3-month ORR of 26.7% of combined cryoablation and PD-1 blockade therapy in liver metastatic melanoma. Meanwhile, recent studies showed a response rate of only 4.3% and 8.9% for the treatment of liver metastasis using PD-l blockade monotherapy among patients with cutaneous melanoma [12] and uveal melanoma [14], respectively. These results altogether indicated that adding cryoablation of intrahepatic lesions to PD-1 blockade therapy is an effective way to achieve an enhanced anti-tumor immune response. Generally, the recommended safety margin of thermal ablation for liver metastases is 10 mm, while there are limited data on the ablation of liver metastases from melanoma. Our experience on cryoablation of liver metastatic melanoma suggests that a safety margin of 5 mm is sufficient to achieve a high rate of complete ablation, which is, therefore, adopted in our study.

The general response rate of this combined strategy was still lower than 30%, and identifying the patients who can benefit from this combined treatment is undoubtedly important. Our study showed that metastatic organs  ≤ 2, pre-treatment LDH level  ≤ 300 U/L, and the diameter of ablated lesion  ≤ 30 mm were key factors that predict better treatment response and survival. The number of metastatic organs potentially indicates the malignant degree of the tumor and has been reported to be correlated with the survival of patients receiving PD-1 blockade therapy [15]. Elevated lactate dehydrogenase (LDH) is a known predictive and prognostic factor for poor outcomes in patients with metastatic melanoma [16]. A very counterintuitive and interesting finding of this study was that the diameter of ablated lesion  ≤ 30 mm predicted better treatment response, while it used to be thought that larger lesion ablated can release more tumor-specific antigens and theoretically lead to a higher chance for inducing an anti-tumor immune response compared to ablation of small lesions [17]. Urano et al. [18] compared the immunological effect of cryoablation of three nodes with cryoablation of only a single node in a mice model with liver metastatic tumors and found that cryoablation of a single node could activate anti-tumor immune response while cryoablation of three nodes did not. Both Urano’s pre-clinical results and the finding of our study suggested that the phenomenon of high zone tolerance [19] may exist for cryoablation of liver metastasis. On the other hand, theoretically, ablation of large intrahepatic tumors can cause high level of inflammation, which is consistent with our observation that cryoablation of tumor  > 3 cm lead to greater increases in both serum level of NLR (7.21 vs 3.59) and IL-6 (15.40 pg/ml vs 8.62 pg/ml) after treatment as compared with cryoablation of tumor  ≤ 3 cm. The differences were prominent, although statistical significance had not been achieved partly due to the limitation of sample size. The effects of neutrophils and IL-6 on tumor immune invasion had been demonstrated in recent years [20]. Domenico et al. [21] found that increased NLR after treatment is associated with an increased neutrophil population, with polarization to the N2 phenotype, and may be the basis for the negatively prognostic role of NLR among patients with metastatic melanoma receiving immunotherapy. Their another work in investigating the prognostic role of IL-6 among patients with advanced cutaneous squamous cell carcinoma found both high serum levels of IL-6 at baseline and increase after PD-1 blockade immunotherapy predict unfavorable prognosis [22]. The prominent increase in NLR and IL-6 levels after cryoablation of large intrahepatic tumors may also account for the phenomenon of poor prognosis in our study. Fine selections, based on both the baseline characteristics of patients and the size of the intrahepatic lesion for cryoablation, are essential to achieve a high response rate in terms of combined PD-1 blockade and cryoablation therapy.

The previous studies exploring the sequencing of combined PD-1 blockade therapy and locoregional therapies suggested that administration of PD-1 antibodies before local treatment may achieve better treatment outcomes, as this can ensure that the immune cells are “prepared” for the upcoming immunological effect of local treatments [23]. In recent years, there were evidences in favor of administration of anti-PD-1 antibodies after local treatment, which facilitated the expansion of the intratumoral polyfunctional intratumoral CD8 + T cells and induction of a potent abscopal effect [24]. In our study, although there was a trend that the administration of PD-1 blockade agents after cryoablation was correlated with superior ORR, the correlation was insignificant (*P* = 0.096); besides, its association with OS was also insignificant (Fig. 2D). More efforts are needed to explore the sequence and timing of each intervention for achieving optimal anti-tumor immune response.

It was interesting to find that CD19 + lymphocytes in peripheral blood could be an important marker of response among patients receiving combined therapy. Sirui et al. [25] found that STING-induced regulatory B cells (Bregs) can compromise NK function in cancer immunity, which provides us a clue that the CD19 + cells in our study may reflect the abundance of regulatory B cells (Bregs) of patients. More studies are needed to explore the impact of Bregs on the anti-cancer immunity of melanoma patients with liver metastasis.

The current study had several limitations. First, the nature of the retrospective study design may cause potential patient selection bias. Second, the sample size of included participants is relatively small, partly due to the rarity of liver metastatic melanoma patients, which may restrict the weight of the evidence. Third, a small proportion of patients received additional systemic chemotherapy during the combination treatment, although our analysis found no correlations between the intervention and ORR/OS of patients, this factor may confound the efficacy of PD-1 blockade and cryoablation combination therapy. Currently, a phase-II single-arm prospective trial (CryoCheck-001; ChiCTR2200061591) to validate the safety and efficacy of cryoablation combined with sintilimab (PD-1 antibody) in the treatment of liver metastases of melanoma is under conduction in our cancer center, which may bring more advances in this area in the future.

### Supplementary Information

Below is the link to the electronic supplementary material.

Figure S1. Treatment response and serum immune correlative results during the combination treatment (TIF 2882 KB)

Table S1. Comparison of cryoablation for different tumor sizes in the first cycle of combination treatment (DOCX 14 KB)

## Abbreviations

CI: Confidence interval
CT: Computed tomography
ECOG: Eastern Cooperative Oncology Group
HR: Hazard ratio
LDH: Lactate dehydrogenase
MRI: Magnetic resonance imaging
ORR: Overall response rate
OS: Overall survival
PD-1: Programmed death-1
