# Supplementary figures and images for: Size selection of intrahepatic lesions for cryoablation contributes to abscopal effect and long-term survival in patients with liver metastatic melanoma receiving PD-1 blockade therapy
Source: Cancer Immunol Immunother. 2024 Mar 2;73(4):68. doi: 10.1007/s00262-024-03637-1 (PMC10908608; doi:10.1007/s00262-024-03637-1)

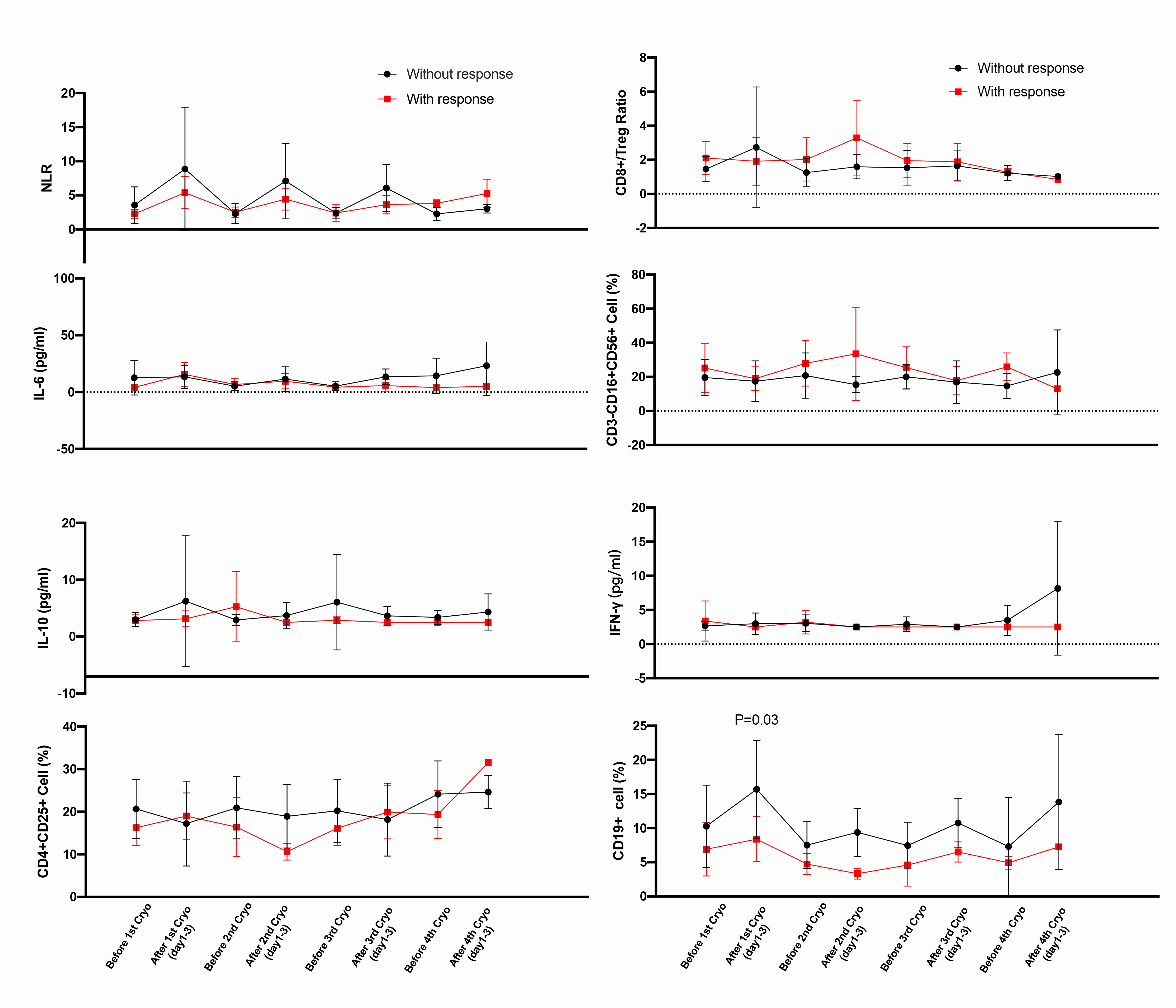

Supplement: Supplementary file 1 — Figure S1. Treatment response and serum immune correlative results during the combination treatment (TIF 2882 KB) [file 262_2024_3637_MOESM1_ESM.tif]
