# Supplementary material for: Size selection of intrahepatic lesions for cryoablation contributes to abscopal effect and long-term survival in patients with liver metastatic melanoma receiving PD-1 blockade therapy
Source: Cancer Immunol Immunother. 2024 Mar 2;73(4):68. doi: 10.1007/s00262-024-03637-1 (PMC10908608; doi:10.1007/s00262-024-03637-1)
Supplement: Supplementary file 2 — Table S1. Comparison of cryoablation for different tumor sizes in the first cycle of combination treatment (DOCX 14 KB) [file 262_2024_3637_MOESM2_ESM.docx]

**Table S1. Comparison of cryoablation for different tumor sizes in the first cycle of combination treatment**

| Category | Diameter of ablated lesion ≤30 mm, n(%) | Diameter of ablated lesion >30mm, n(%) | P value |
| --- | --- | --- | --- |
| Diameter of the intrahepatic lesion (mm; median, range) | 21.5 (6-48) | 48 (20-98) | 0.001^β^ |
| Diameter of the intrahepatic lesion (mm) |  |  | <0.001* |
| ≤30 | 24 (80.0) | 2 (13.3) |  |
| >30 | 6 (20.0) | 13 (86.7) |  |
| Number of intrahepatic metastases |  |  | 0.456* |
| ≤3 | 8 (26.7) | 2 (13.3) |  |
| >3 | 22 (73.3) | 13 (86.7) |  |
| Number of cryoablation needles |  |  | 0.026* |
| ≤2 | 26 (86.7) | 8 (53.3) |  |
| >2 | 4 (13.3) | 7 (46.7) |  |
| Abdominal bleeding |  |  | 0.106* |
| Absent | 30 (100.0) | 13 (86.7) |  |
| Present | 0 (0.0) | 2 (13.3) |  |

*Fisher’s Exact test; β Independent samples t-test.
